# Carcinogenic and non-carcinogenic risk assessment of consuming metal-laden wild mushrooms in Nigeria: Analyses from field based and systematic review studies

**DOI:** 10.5620/eaht.2023013

**Published:** 2023-06-30

**Authors:** Kanayo Stephen Chukwuka, Samuel Oluwasegun Adesida, Chibuisi Gideon Alimba

**Affiliations:** 1 Department of Botany, University of Ibadan, Nigeria; 2 Department of Zoology, University of Ibadan, Nigeria

**Keywords:** Carcinogenic health risk, metal pollution, mushrooms, non-carcinogenic health risk, Nigeria, risk assessment

## Abstract

This study investigated the potential health risk associated with the consumption of metal-laden mushrooms in Nigeria. Concentrations of Pb, Cd, Cr, Cu, Ni, Zn and Al in wild mushrooms collected from the Nigerian environment were measured using atomic absorption spectrometer. Also, systematic analysis of articles on metal accumulation in mushrooms from Nigeria were obtained from scientific databases. Using hazard model indices, the metal concentration in mushrooms were evaluated for their potential carcinogenic and non-carcinogenic health risk when consumed by adults and children. Zn and Cd, respectively, had the highest and lowest mean concentrations (mg kg^-1^) in the analysed mushrooms from the field study, while Fe and Co, respectively, had the highest and lowest mean concentrations (mg kg^-1^) in the systematically reviewed articles. In the field study, the percentage distribution of THQ of the heavy metals greater than 1 was 0% and 42.85% for adults and children respectively. While for the systematic study, 30% and 50% of the heavy metals for adults and children respectively exceeded the limit of 1. The hazard indices obtained from both the systematic and field studies for both age groups were all >1, indicating significant health risk. The findings from both the systematic and field studies revealed that consuming metal-laden mushrooms by adults and children increases the carcinogenic risk to Cd, Cr, and Ni since they exceeded the acceptable limit of 1E-04 stated by USEPA guideline. Based on the findings from the systematic and field studies, it suggests that consuming mushrooms collected from metal polluted substrates increases carcinogenic and non-carcinogenic health risk among Nigerians.

## Introduction

Edible mushrooms are globally acknowledged as potent sources of proteins, vitamins, fibre, minerals and low cholesterol [1]. In addition to the nutritional value of mushrooms, they possess various therapeutic properties. Some of the bioactivities they exhibit include anti-inflammatory, antioxidant, antiviral, antibacterial, antifungal, anticancer, immuneregulatory, anti-diabetic and cholesterol-lowering activities [2, 3, 4, 5]. Over ten (10) different species of mushroom, with high nutritional and medicinal importance, are commonly consumed in Nigeria [6, 7]. However, despite the nutritional and health benefits derived from mushrooms, their ability to readily accumulate pollutants, most importantly metals, from contaminated environment and subsequently bio-concentrate these pollutants in tertiary consumers including humans, is increasingly being reported around the world [5, 6, 8-12]. Human exposure to toxic chemicals through the consumption of mushrooms has been suggested a potential source of direct metal accumulation, and this poses great risk to human and wildlife (herbivores) health and survival [9-11, 13]. It is important to increase research output to enable unraveling the paucity of information which exist on the plausible health effects associated with consuming mushroom laden with heavy metals by organisms at the higher trophic levels.

Mushrooms possess effective mechanisms that enhance adequate absorption and translocation of toxic chemicals from contaminated sites when compared with many other plants growing in the same or similar environment [14-16]. Through the mycelium, mushrooms absorb toxic metals from the substrates into the fruiting bodies [11, 12, 17]. Colak et al. [18] reported that patients who consumed poisoned mushrooms collected from the wild expressed nausea and alteration in aspartate aminotransferase and alanine aminotransferase. Furthermore, in 2010, an incidence of lead poisoning in Zamfara State, Nigeria which claimed the lives of over 400 children within seven months [19], was associated with the consumption of lead-laden food crops harvested from metal contaminated soil between 2010 and 2013 [20]. Moszynski [21] had earlier reported that the Zamfara State lead poisoning epidemic may be connected with exposure to soil Pb level which exceeded 100,000 mg kg^-1^. Considering that the consumption of metal-laden mushroom is one of the many ways via which heavy metals gain entry into the human body system [22], it becomes imperative to conduct health risk assessment of consuming metal laden mushrooms. Olatunji-ojo et al. [11] cultivated Pleurotus ostreatus (mushroom) in Pb-contaminated rice straw and observed high accumulation of Pb in the mushroom fruit bodies. Aqueous extract of the harvested Pb laden mushroom was fed to mice and it resulted in Pb accumulation in the testes with subsequent expression of abnormal sperm morphology, abnormal testes pathology and bone marrow cell micronucleus formation. These are biomarkers of somatic and germline genetic and cellular damage, and may invariably suggest pathophysiological disorders including cancer initiation and progression [23].

According to the Nigeria Cancer Statistics, about 102,100 new cancer cases were diagnosed annually, while at least 71,600 mortalities occur annually, due to various stages of cancer progressions [6]. Due to increase in hunger (high cost of food and feeding) in Nigeria and many other countries (especially developing countries) of the world, many populations readily depend on mushrooms harvesting from the wild to avert hunger and nutritional deficiencies [24]. It is hypothesized that the act of ignorantly consuming wild mushrooms due to intense hunger or its uninformed use in ethnomedicine can increase exposure to heavy metals and subsequently pathophysiological disorders. Therefore, this study aimed at increasing knowledge on the possible carcinogenic and non-carcinogenic risks associated with human consumption of metal-laden mushrooms in Nigeria. The specific objectives include:

I. Heavy metal analysis of mushrooms collected from the wild,
II. Using Preferred Reporting Items for Systematic Reviews and Meta-Analyses (PRISMA) to collect data of studies on metal accumulation in mushroom from the Nigerian environment.
III. Estimation of carcinogenic and non-carcinogenic health risk associated with the consumption of mushrooms using data generated for different metals in the field and systematic review studies.

Risk assessment models that defined the tolerable intake dose and carcinogenic and non-carcinogenic risk as posited by universal standards [25-28] are employed in the assessment of carcinogenic and non-carcinogenic risk herein reported.

## Materials and Methods

### Mushroom collection and identification

**Figure 1.**
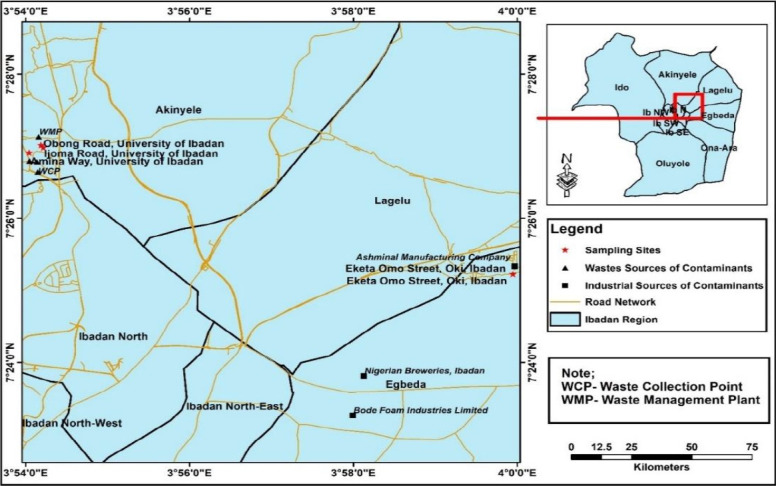
Map of study area showing the sampling site

Six wild mushroom species were collected between July and September, 2021 from two local governments in Ibadan, Southwestern Nigeria (Fig. 1). Studies in this part of the country have revealed the ethnomycological use of wild edible and medicinal mushrooms by majority (about 87.2%) of the population [7, 24, 29]. The mushrooms were mainly collected from decaying logs which served as substratum for their growth. The mushrooms were identified with their morphological characteristics in accordance with taxonomic classifications by Hall et al. [30], Loyd et al. [31], Mcknight and Mcknight [32] and Schwab [33]. Details of the mushroom collections are presented in Table 1.

### Digestion of the collected mushroom samples and metal analysis

The mushrooms were air dried in the laboratory for 14 days and ground into powdered form using Cutting Boll Mill. The nitric-perchloric acid digestion method was employed in accordance with AOAC recommendation [34]. Blanks were prepared as quality control samples by applying 5 mL of concentrated nitric acid into empty digestion flasks and processed through the entire analytical method to ensure accuracy of data [34]. Afterwards, 0.5 grams of each air-dried sample was transferred into a 100 mL beaker and 5 mL of an acid mixture containing nitric (HNO_3_) and perchloric (HClO_4_) acids in the ratio 2:1 was added. The sample was heated at 120 – 150℃ for 45 minutes under a fume-hood in order to initiate the reaction process. The digestion process was completed when a clear solution was obtained. The solution was allowed to cool before further filtering through Whatman No. 42 filter paper and diluting with distilled water to 25 mL. The digested mushroom samples were then measured in triplicates to determine Pb, Cd, Cr, Cu, Ni, Al, and Zn concentrations using atomic absorption spectrophotometer (AAS; Buck Scientific model 210/211 VGP, East Norwalk, USA).

### Systematic review analysis of metal accumulation in mushrooms from the Nigerian environment

#### Search strategy

**Figure 2.**
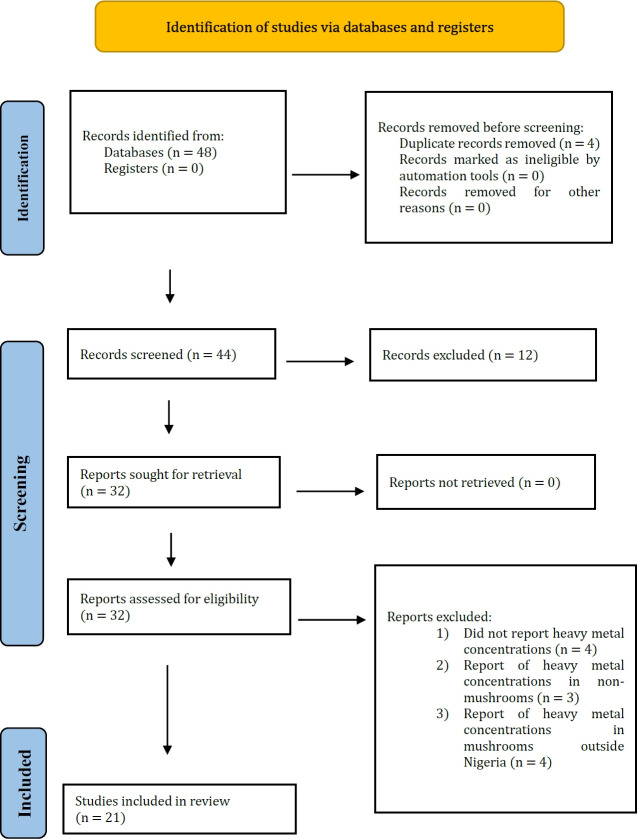
PRISMA flow chart for the systematic review report on heavy metal concentrations of mushrooms in Nigeria

Published scientific articles that reported toxic metals in mushrooms from the Nigeria environment were searched and selected from scientific databases: Google Scholar, PubMed, Scopus, Directory of Open Access Journals (DOAJ) Web of Science, in accordance with PRISMA (preferred reporting items for systematic reviews and meta-analysis) guidelines (Fig. 2). Articles published between the year 1980 and 2022 were retrieved. The following keywords, phrases and clauses: “heavy metals in mushrooms from Nigeria”, AND “metal in mushrooms from Nigeria”, AND “metal contamination of mushroom from Nigeria”, AND “names of the individual metals (Pb, Cr, Cd, As, Cu etc.) contaminated mushrooms from Nigerian environment”, AND “Mushrooms collected from contaminated Nigerian environment” were used for article search. At the end, the papers were screened and re-checked in order to eliminate duplicate and unrelated papers.

#### Inclusion and exclusion criteria

The inclusive criteria include:

1. articles reporting concentration of heavy metals in mushrooms collected from Nigeria,
2. articles published in English language and in peer-reviewed journals,
3. articles must clearly present data of metal concentrations as mean or range.

Papers not included in this study were screened out based on the following criteria:

1. articles reporting heavy metal concentrations in non-mushroom consumables,
2. articles reporting heavy metal concentrations in mushrooms but not collected from Nigeria,
3. articles with duplicate content,
4 articles written in other languages other than English,
5. articles with only abstract available (Book of abstract from conferences).

#### Statistical analysis

The concentration of metals obtained from the mushroom collected from the field and the systematic review data were analyzed using GraphPad prism version 8.0™ and the results presented as Mean ± SD. These data were used to calculate the carcinogenic and non-carcinogenic health risk using the various human health risk standard models.

#### Health Risks Assessment

The consumption of metal laden mushrooms suggests that humans are exposed to metals via oral exposure route. Hence, the carcinogenic and non-carcinogenic health risks calculated for Nigerians that consume mushrooms containing metals were based on the estimated daily intake (EDI) of the toxic metals via ingestion of mushroom (oral route) in accordance with the United States Environmental Protection Agency [35] model using the threshold values. Target hazard quotients and Hazard index were calculated and the values used to evaluate the potential non-carcinogenic health risk for adult and children who may be consuming metal laden mushroom.

#### Estimated Daily Intake (EDI)

Daily intake of metals depends on both the metal concentration in food and the daily food consumption. Also, the body weight of exposed person can affect ability to tolerate the metal. EDI is a model that estimates or evaluates the rate of transfer of metals from consumed food substance into the human body [4]. EDI values for each metal is calculated using the equation below:


(1) $$\mathrm{EDI}=\frac{\mathrm{Concentration}\;\mathrm{of}\;\mathrm{metal}\;\times \;\mathrm{Daily}\;\mathrm{intake}\;\mathrm{of}\;\mathrm{food}\;(0.03\;\mathrm{kg}/\mathrm{person})}{\mathrm{Average}\;\mathrm{body}\;\mathrm{weight}\;}$$

According to Joint FAO/WHO Expert Committee on Food Additives [36], average body weight for adult is considered as 70 kg, while that for children is 24 kg. Also, the average daily intake of mushroom is accepted as 0.03 kg per person [4]. The concentration of metals obtained from the analyses of mushrooms collected from the field and systematic review (see Tables 2 and 3 respectively) were used for the EDI evaluation and other related estimations where necessary.

#### Target Hazard Quotient (THQ)

Target Hazard Quotient describes the ratio of exposure to toxic substance and the reference dose which is the highest concentration at which no adverse health effects are possible or envisaged [6]. THQ estimated value explains the probability of non-carcinogenic health risk of consuming the mushrooms. It is estimated from calculating the ratio of the determined dose of a pollutant to a reference level considered toxic [4]. When the estimated value of THQ is < 1, then non-carcinogenic health effects are not expected. When THQ is >1, there is tendency that adverse health effects may occur [4, 8]. THQ of the heavy metals were estimated using the formula:


(2) $$\mathrm{THQ}=\frac{\mathrm{Estimated}\;\mathrm{Daily}\;\mathrm{Intake}\;\mathrm{of}\;\mathrm{metal}}{\left({\mathrm{RfD}}_{\mathrm{ingestion}}\right)}$$

RfD_ingestion_ represents oral reference dose for each specific metal: Pb = 0.004, Cd = 0.001, Cr = 0.003, Ni = 0.02, Zn = 0.3, Cu = 0.04, Al = 1, Co = 0.0003, Mn = 0.14, Fe = 0.7 [26, 37].

#### Hazard Index (HI)

Hazard Index is calculated from estimating the sum of individual values of the THQ of the studied elements in each mushroom sample. When HI value is higher than 1, it is considered a substantial risk and danger to health [6, 38]. The HI index is calculated as follows:


(3) $$\mathrm{HI}=\sum_{THQs}^{,}n$$

Where; THQ is the target hazard quotient estimated for each toxic element (n)

#### Carcinogenic Risk (CR)

Carcinogenic Risk is a measure of the likelihood of expressing or developing cancer during a lifetime as a result of exposure to carcinogens [26, 38]. The CR value below 1x10-4 is considered an acceptable risk, while values higher than 1x10-4 imply an increased risk of carcinogenic effect associated with exposure to the carcinogens [26]. The CR is calculated from the equation below:


(4) CR=EDI×Csf

Csf represents the oral slope factor of specific carcinogen: Pb = 0.0085, Cd = 6.3, Cr = 0.5, Ni = 0.84 [4, 26].

## Results

### Concentration of heavy metals

**Figure 3.**
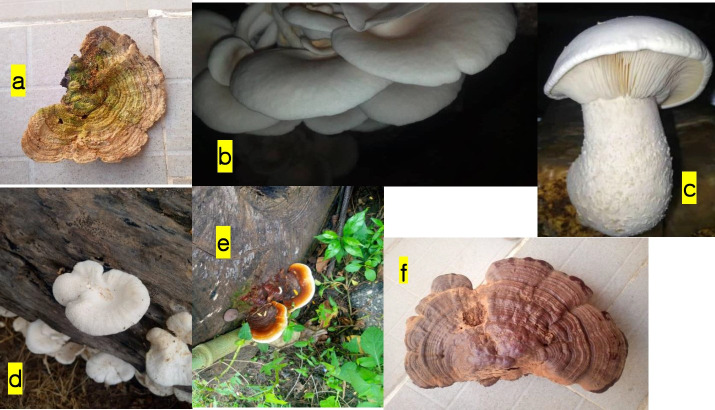
presents the harvested mushrooms evaluated for heavy metal accumulation: (a) *Lenzites betulina*; (b) *Pleurotus ostreatus*; (c) *Calocybe indica*; (d) *Pleurotus pulmonarius*; (e) *Ganoderma sessile*; (f) *Ganoderma applanatum*

Table 2 presents the mean concentrations of the analysed metals observed in the collected mushroom samples. All the collected mushrooms accumulated varying concentrations of the various heavy metals. *L. betulina* accumulated the highest mean concentration for most of the studied metals including; Cd, Cu, Ni, Zn, and Al while *P. ostreatus* recorded the lowest mean concentration for Pb, Cu, Ni and Zn. The concentrations of Zn and Cd were the highest and lowest respectively in all analysed mushrooms. Fig. 3 presents morphology of the representatives of analysed mushrooms.

### Estimated daily intake

Human health risk assessment of heavy metal contamination of wild mushrooms revealed that Zn and Cd had the highest and lowest mean concentrations in mushrooms obtained from the field study. While Fe and Co had the highest and lowest mean concentration according to the data generated from the analysis of the systematic review. Table 3 presents the estimated daily intake (EDI; µg/kg/day) of metals in mushrooms collected from the field study and calculated for both children and adults. The EDI (µg/kg/day) for Pb range in adult is 2.71 – 3.57 and 7.91 – 10.41 in children. Also, for Cd is 0.46 – 0.69 and 1.35 – 2.03 for adult and children respectively. Cr is 1.26 – 2.06 and 3.66 – 6.01 for adult and children respectively, Cu is 1.83 – 7.69 and 5.33 – 22.44 for adult and children respectively, Ni is 0.72 – 2.48 and 2.10 – 7.23 for adult and children respectively, Zn is 9.27 – 57.60 and 27.03 – 168.00 for adult and children respectively, and Al is 1.59 – 3.47 and 4.65 – 10.13 for adult and children respectively. For the children group, the EDI of Cd in all the studied mushrooms exceeded the Permissible Tolerable Daily Intake (PTDI) set by Joint FAO/WHO Expert Committee on Food Additives [36] (Table 3).

### Target hazard quotient

Table 4 presents the target hazard quotient (THQ) of the analysed metals for adult and children. The THQ for Pb is in the range 0.68 – 0.89 and 1.98 – 2.60 for adults and children respectively, Cd in the range 0.46 – 0.69 and 1.35 – 2.03 for adults and children respectively, Cr in the range 0.42 – 0.69 and 1.22 – 2.00 for adults and children respectively, Cu in the range 0.05 – 0.19 and 0.13 – 0.56 for adults and children respectively, Ni in the range 0.04 – 0.12 and 0.11 – 0.36 for adults and children respectively, Zn in the range 0.03 – 0.19 and 0.09 – 0.56 for adults and children respectively, Al in the range 0.0016 - 0.0035 and 0.0047 – 0.01 for adults and children respectively. *Lenzites betulina* recorded the highest THQ for most of the heavy metals for adults (Pb, Cd, Cu, Ni, Zn, Al) and children (Cd, Cu, Ni, Zn, Al) while the lowest THQ for most of the analyzed heavy metals for adults and children (Pb, Cu, Ni, Zn) was recorded in *Pleurotus ostreatus* (Table 4).

### Hazard Index

**Figure 4.**
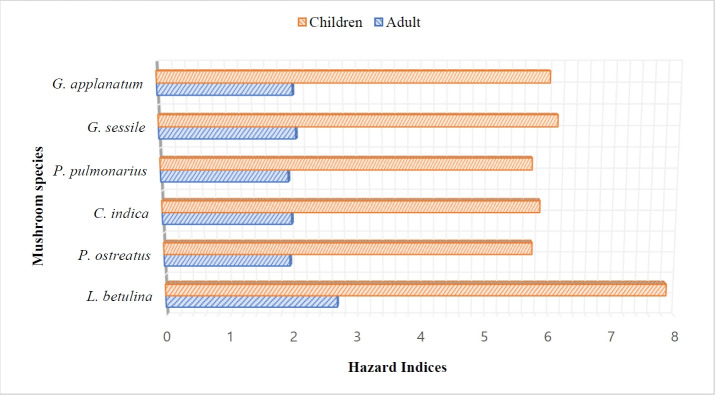
showing the hazard indices of the analysed mushroom sampled from the southwest region of Nigeria

The Hazard indices of the analysed mushrooms collected from the filed study ranged from 1.96 – 2.69 in adults and 5.71 – 7.85 in children (Table 4). The highest and lowest values for both of these groups were recorded in *L. betulina* and *P. ostreatus* respectively (Fig. 4). The HI of all the mushrooms for both adult and children exceeded the threshold limit of 1. Furthermore, HI estimated for children is higher than that of the adults in all the analysed mushroom samples (Fig. 4).

### Carcinogenic risk.

The Carcinogenic Risk (CR) of the analysed heavy metals in the mushrooms collected from the field is presented in Table 5. The carcinogenic risk of Al [39], Cu [40] and Zn [41] was not estimated in this study since there is no evidence of their classification as carcinogens yet. According to the analysed data on Table 5, the CR for the heavy metals in adults and children respectively ranged as follows: Pb (2.30–3.04E-05 and 6.72–8.85E-05), Cd (2.92–4.37E-03 and 8.51E-03–1.27E-02), Cr (6.3E-04–1.03E-03 and 1.83–3.01E-03), and Ni (6.05E-04–2.08E-03 and 1.76–6.07E-03). The highest values for Cd and Ni for both adults and children were estimated in *L. betulina*. On the other hand, the lowest values for Pb and Ni in both adult and children were estimated for *P. ostreatus* while the lowest value for Cd in both adults and children was estimated for *P. pulmonarius*.

### Systematic review

#### Inclusion of studies

A total of 48 articles were retrieved from the scientific databases. 4 of them were excluded due to duplication (Fi g. 2). The papers were screened for eligibility and 23 papers were further considered unsuitable. 21 articles fulfilled the req uired criteria for selection and were eventually included in the study (Fig. 2).

#### Concentration of heavy metals

The mean concentration (mg kg^-1^) of heavy metals in the reviewed selected articles are in the order: Fe (255.98)>Cu (50.39)>Zn (37.72)>Mn (19.89)>Cr (10.25)>Al (9.15)>Cd (8.45)>Pb (6.49)>Ni (5.16)>Co (3.29) (Table 6).

#### Health risk assessment

Analyses of EDI, THQ, HI and CR in adults and children estimated using the generated mean of the respective heavy metals in the reviewed articles were presented in Table 7. Estimated Daily Intake (EDI) analysis of heavy metals from the systematic reviewed articles ranged from 1.41–109.70 µg/kg/day for adults and 4.11–319.98 µg/kg/day for children. The lowest and highest values were respectively recorded for Co and Fe for both adults and children. The THQ analysis for heavy metals in the selected reviewed articles ranged from 0.00392–4.7 for adults and 0.011-13.66 for children. Al and Co respectively recorded the lowest and highest values for both groups. In the adult group, the THQs for Cd, Cr and Co exceeded the safe limit of 1 while the THQs for Pb, Cu, Ni, Zn, Al, Mn and Fe were within the safe limit. Albeit for the children group, the THQs for Pb, Cd, Cr, Cu and Co exceeded the safe limit of 1, while THQs for Ni, Zn, Al, Mn and Fe were within the safe limit. The HI for the adult (11.4) and children (33.2), which described the sum of all THQs for the analysed heavy metals, exceeded the acceptable limit of 1 for both the adults and children categories. The carcinogenic risk (CR) of the metals ranged from 2.36E-05 – 2.28E-02 for adults and 6.89E-05 - 6.65E-02 for children (Table 7). The CR for Cd, Cr and Ni exceeded the acceptable limit of 1E-06 – 1E-04 while CR for Pb was within the limit for both groups.

## Discussion

### Heavy metal accumulation in mushrooms

Heavy metals are the oldest ubiquitous chemicals globally studied for their high levels of deleterious effects on public health [60-62]. In the scientific publication “A silent epidemic of environmental metal poisoning”, Nriagu [63] stated that “it is incontestable that each environmental compartment has a limited carrying capacity for metal pollution and with enough time, the current rates of metal inputs will become stressful to many ecosystems”. After 30 years of the position, numerous studies abound in literature that reported high metal contamination of most aquatic and terrestrial environments of the world. Also, many other scientific reports showed that biota (both plants and animals) inhabiting these ecosystems were highly laden with different metals [64-68]. Macromycetes (mushrooms) are hyper-accumulators of heavy metals. This informs its use in the cleaning up of contaminated environment [69]. However, the health risk associated with the possibility of consuming such mushrooms had gained little attention. According to Oyetayo [7], Nigerian traditional herbalists and rural dwellers exploit wild mushrooms for two main reasons: as source of nutrition (food) and biological active molecules which contains pharmaceuticals that can aid specific body functions (medicine). Some of the mushrooms used for medicinal purposes are consumed according to prescriptions to aid the treatment of various ailments. In other instance, the extracts of the fruiting bodies and mycelia of the mushrooms are utilized in the ethnomedicinal practices. The ethnomedicinal use of the wild mushrooms examined in this study has been documented by a number of Nigerian scientists [7, 70-72]. According to reports, the Igbo population residing in the Southeastern Nigeria utilize *G. applanatum* for anti-oxidative, antihypertensive, and anti-diabetic purposes, while *G. sessile* is used to treat neoplasia and arthritis [70]. Also, the ethnomedicinal use of wild mushrooms was reported to be prevalent among the Igalas of the Northcentral Nigeria [71]. Considering that consuming metal laden mushrooms for ethnomedicinal purpose may increase metal accumulation in humans, this justifies the evaluation of both the edible and ethnomedicinal mushrooms collected from the wild for their carcinogenic and non-carcinogenic health risks in the study herein presented.

All the mushrooms collected from the wild had varying concentrations of heavy metals ranking in the order: Zn > Cu > Pb > Al > Cr > Ni > Cd (Table 6). Also, data generated from the systematic review studies on metal accumulation in mushroom collected from the Nigerian environment using PRISMA (Fig. 2) presented the mean concentrations of the metals in the order: Fe > Cu > Zn > Mn > Cr > Al > Cd > Pb > Ni > Co (Table 3). Fe, Cu, Mn, Zn, and Cr are essential micronutrient that forms components of many important enzymes and cofactors required for adequate functioning of the body system both in plants and animals. These metals are important actors in DNA replication (synthesis), protein synthesis, improving and fostering healthy immune systems and repair and healing of damaged tissues [73, 74]. Howbeit, they are required in trace quantities by animals and plants, but when accumulated in the body of organisms above the permissible limit, they may induce abnormal pathophysiological processes in the body.

Metal accumulation in mushrooms had been linked to chitin, chitosan, glucan and amino polysaccharide present in the cell wall. These molecules readily absorb metals from the substrates of the mushroom and pass them to the fruiting bodies. Metals may bind to the amine-N group of the chitin which acts as the nucleation site for the absorption of metals into the mushrooms [69, 75]. This assertion is supported by the report that Cd accumulation in *Neurospora crassa* was through the N-acetylglucosamine polymer and chitin present in the fungal cell wall [76]. Also, these molecules were isolated from the cell wall of white rot fungus (*Coriolopsis polyzona*) in an attempt to understand their role in bioaccumulation [77]. In the laboratory settings, the isolated molecules when used as biocatalyst successfully removed endocrine disrupting chemicals, nonylphenol, bisphenol A and personal care product ingredient, triclosan, from contaminated substrates [77]. Furthermore, there are some plasma membrane transporters which are similarly involved in metal ion uptake from the soil or substratum into the cytosol of the root cells. For instance, the OsNRAMP6 transporter, which is localized to the plasma membrane and expressed in the shoot system, serves as both Fe and Mn transporter in yeast [78]. These evolutionary acquired mechanisms that enables the absorption of essential metals by mushrooms aims at enhancing proper growth and development of mushrooms [73]. However, owing to the valence of non-essential metals like Pb and Cd being similar to the essential metals, most of these mushrooms rarely discriminate between absorbing essential and non-essential metals [73].

Differences in the rate of absorption of metals by the mushroom species (in morphological part of fruiting body and age of mycelium) and the composition of the metals in the substrates, rate of metal accumulation in mushrooms show variations [6, 14, 59], may possibly explain why *Lenzites betulina* (Fig. 3a) accumulated the highest concentrations for most of the studied metals; Cd, Cu, Ni, Zn, Al, while *Pleurotus ostreatus* (Fig. 3b) accumulated least concentrations for most of the analysed heavy metals; Pb, Cu, Ni, and Zn among the harvested mushrooms (Table 2). Metal accumulation in mushrooms is a global menace that poses danger to public health. For instance, Świsłowski et al. [79] conducted a bibliometric analysis of 200 publications on metal accumulation in mushrooms from the European countries, between 2001 and 2016. The authors observed that 492 species of mushrooms (wild-growing and cultured) common in 26 European countries accumulated majorly Cd, Cu, Fe, Pb, and Zn. More than 50% of the reviewed mushrooms are edible and the studies were primarily from Turkey, Poland, Spain, and the Czech Republic [79]. Since it is now well established that toxic metals and other chemical elements accumulates in mushrooms, it is imperative to conduct the risk assessment to human health that may occur when metal-laden (importantly Cd, Hg and Pb) mushrooms are consumed.

### Risk assessment to human health from the consumption of metal-laden mushrooms

Collection and consumption of wild mushrooms are cultural and social activities in most countries in Europe, Asia, and in the United States [80]. Meanwhile, mushroom consumption is a major source of food and raw material for ethnomedicine in Africa [81]. Therefore, exposure to heavy metals and other contaminant through mushroom consumption is imminent. Furthermore, long time consumption of metal-laden mushrooms may be detrimental to human health. Standardize models including Estimated Daily Intakes (EDI), Target Hazard Quotient (THQ), Hazard Index (HI) and Cancer Risk (CR) were used to assess the potential risk associated with the consumption of metal-laden mushroom among Nigerians. The EDI of Pb, Cd, Cr, Cu, Zn, Ni and Al by a 70kg adult and 24kg child who consume 30g of mushrooms daily as calculated from the study herein were below the permissible tolerable daily intake (PTDI) [36], except for EDI of Cd in all the analyzed mushroom samples by children which exceeded the PTDI limit. This observation has been similarly observed by Igbiri et al. [6] and Nnorom et al. [14] from Nigeria, except that Igbiri et al. [6] observed safe levels for Cd for children. The estimated THQ of all the heavy metals in the mushrooms consumed by adults were below 1 while that of Pb, Cd and Cr were above 1 in the children group suggesting a high risk of toxicity from oral exposure to these heavy metals by children. The THQ for Cd, Cr and Co exceeded the limit of 1 in the systematic analysis, hence, suggesting adverse health impact of these metals in mushrooms from Nigeria.

The HI, an index that sums up the individual values of THQ of the analyzed metals in a mushroom sample, is used to investigate the human health risk linked with exposure to multiple elements in a mushroom sample [5]. The HI observed for both the field and systematic analyses exceeded the acceptable limit of 1. This suggests a significant threat to the health of humans who frequently consume mushrooms in Nigeria. This finding had similarly been observed by Igbiri et al. [6] among Southern Nigerians. CR estimation in one per million (1 × 10^-6^) suggests that in any population of 1 x 10^6^ exposed people, there is possibility that at least a cancer case may be expected [6]. CR higher than 10^-4^ indicates an increased risk of the carcinogenic effect caused by the contaminant [4]. The CR estimated for Cd, Cr, and Ni, in both the field study and data generated from the systematic review, exceeded the tolerable limit of 1x10-4 while the CR of Pb was within the satisfactory range of 1x10^-6^ – 1x10^-4^. The distinctly high value of CR for Cd, Cr and Ni may suggest they pose significant carcinogenic risk to mushroom consumers in Nigeria.

Metal accumulation in the mushrooms collected from the field and those from the systematic review is mostly due to high metal concentrations in contaminated soils/substrates. In Nigeria, solid wastes which usually serve as substrates for wild mushroom growth, contain mixtures of electronic wastes, hospital wastes, municipal wastes, industrial wastes and agricultural wastes [82, 83]. These wastes which are metal-laden are indiscriminately co-disposed in the Nigerian environment [84, 85, 86]. Since the spores from mushrooms (fungi) are airborne, the solid wastes provide good environment for their germination and growth. Although, the analyzed metals in most of the collected mushrooms were below standard permissible limits [36] (Table 2), however, it is important to emphasize that heavy metals readily accumulate in both soft and hard tissue part of the body once they gain entry into the body [87]. It is possible they reach threshold concentrations in the body and induce deleterious health effects. Furthermore, in vitro cytotoxicity and genotoxicity study using WIL-2 NS cells [82], and in vivo genotoxicity study using *Clarias gariepinus* [88, 89], have confirmed that metals are capable of interacting additively, synergistically and/or antagonistically to elicit greater toxicological effects even when present in low concentrations [90]. The THQ, HI and CR estimated in the study herein suggest threat to quality health of humans who ignorantly may consume metal-laden mushrooms in Nigeria and other part of the world. This is expected to send signal to environmental and health organizations in Nigeria and other part of the globe to avert unforeseen severe health outbreak due to metal poisoning. This suggestion was supported by some laboratory-based research findings wherein male mice fed with aqueous extracts of Pb-laden *Pleurotus ostreatus* for 35 days accumulated Pb in the testes. The accumulated Pb induced decreased testicular weight, increased congestion of the blood vessels, necrosis, and disorganization of the seminiferous tubules [11]. Also, significant increase in abnormal sperm morphology was observed in the caudal epididymis of the exposed mice [11]. Furthermore, the accumulated Pb also increased micronucleated polychromatic (PCE) and normochromatic (NCE) erythrocytes and decrease PCE-NCE ratio in the bone marrow of the exposed mice compared to the control [11, 12].

This observation suggests abnormal reproductive toxicity and increase genome instability [11]. Genome instability is the hallmark of cancer development. Pb, Cd, Al, Hg, and Co observed in the mushrooms from Nigeria (Tables 2 and 3), are among the top metals in the priority list of hazardous substances recommended for their toxicological assessment [91]. In fact, there has not been any known biological relevance of some of the metals in both plants and animals. For instance, considering their ability to cause chromosomal damage and subsequently tumor formation, Cd was classified as group A human carcinogen while Pb was classified as probable human carcinogen (Group 2B) [92]. This further suggests caution and carefulness to avoid any form of ignorance in consuming wild mushrooms and crop plants harvested from soils or substrates laden with metals. It is noteworthy to indicate that all the estimated health risk models in this study were higher for children compared to adults, suggesting that children are more susceptible to carcinogenic and non-carcinogenic health risks from oral exposure to heavy metals. Children are particularly more exposed and susceptible to toxic effects, according to recent WHO [28] report, because they drink more water, consume more food, and breathe more air in relation to their body weight [93]. The anatomic, immunologic, cognitive, and psychologic differences of children in comparison with adults account for their increased vulnerability to health risks [94].

## Conclusions

This study filled some knowledge gaps on the possible health risks associated with consuming metal-laden mushrooms in Nigeria, as it combined both an experimental analysis and systematic review of the heavy metal contents in mushrooms from the Nigerian environment. Although, the THQ of some of the heavy metals in the experimental based analysis of mushrooms and systematic analysis were < 1, the HI revealed a significant health risk as values in both experimental and systematic analyses for adults and children age groups were > 1. The Carcinogenic Risk of Cd, Cr and Ni in adults and children exceeded the tolerable limit set by USEPA with Cadmium posing the greatest threat of cancer in both experimental and systematic analyses in the study herein presented. Based on the alignment in the findings from both the systematic and field analyses, it is suggested that consuming mushrooms collected from polluted substrates increases carcinogenic and non-carcinogenic health risk induced by metals. The deleterious effects may be higher among children than adults. Based on the findings from this study, it is recommended that the National Agency for Food and Drug Administration and Control (NAFDAC) in Nigeria promulgate guidelines for consuming mushrooms in Nigeria. These guidelines will encourage the cultivation of mushrooms in heavy metal and other pollutants free substrates. It will also educate ignorant Nigerians on the hazardousness/health impacts of consuming wild mushrooms harvested from contaminated substrates.

## Figures and Tables

**Table 1.** Collected mushrooms, sample locations, common names, and the ethnomycological use among Nigerians.

Mushroom samples	Sampling site	Sampling coordinates		Common name	Use
		Latitude	Longitude		
*Lenzites betulina*	Amina way, University of Ibadan	70 26’54.6’’N	30 54’2.7’’E	Birch maze-gill, Gilled polypore	Medicine
*Pleurotus ostreatus*	Eketa Omo Street, Oki, Ibadan	70 25’13.4’’N	30 59’57’’E	Oyster	Edible
*Calocybe indica*	Obong Road, University of Ibadan	70 27’0.8’’N	30 54’11.3’’E	Milky white mushroom	Edible
*Pleurotus pulmonarius*	Eketa Omo Street, Oki, Ibadan	70 25’13.4’’N	30 59’57’’E	Lung Oyster	Edible
*Ganoderma sessile*	Amina way, University of Ibadan	70 26’54.6’’N	30 54’2.7’’E	NA	Medicine
*Ganoderma applanatum*	Ijoma Road, University of Ibadan	70 26’58.9’’N	30 54’13.1’’E	Artist’s conk	Medicine

NA – Not applicable

**Table 2.** Mean heavy metal concentrations (mg kg-1) in mushrooms collected from the field in southwest Nigeria

Mushroom samples	Pb	Cd	Cr	Cu	Ni	Zn	Al
*Lenzites betulina*	8.26±0.25 (8.0–8.5)	1.62±0.06 (1.56–1.68)	4.21±0.13 (4.06–4.30)	17.95±0.22 (17.7–18.1)	5.78±0.18 (5.60–5.95)	134.4±2.35 (132.0–136.7)	8.1±0.2 (7.90–8.30)
*Pleurotus ostreatus*	6.33±0.29 (6.0–6.5)	1.13±0.07 (1.08–1.21)	4.81±0.31 (4.50–5.11)	4.26±0.05 (4.2–4.30)	1.68±0.03 (1.64–1.70)	21.63±0.15 (21.5–21.8)	4.28±0.25 (4.01–4.50)
*Calocybe indica*	8.07±0.21 (7.9–8.3)	1.24±0.06 (1.19–1.31)	3.14±0.22 (2.96–3.38)	6.38±0.08 (6.3–6.45)	2.18±0.11 (2.08–2.30)	28.7±0.52 (28.1–29.0)	3.72±0.19 (3.51–3.89)
*Pleurotus pulmonarius*	8.33±0.42 (8.0–8.8)	1.08±0.06 (1.01–1.13)	2.94±0.10 (2.85–3.05)	6.83±0.06 (6.8–6.9)	2.32±0.15 (2.14–2.41)	38.5±0.87 (38.0–39.5)	5.37±0.38 (5.1–5.8)
*Ganoderma sessile*	7.83±0.83 (6.9–8.5)	1.31±0.03 (1.29–1.34)	2.93±0.05 (2.90–2.98)	13.37±0.25 (13.1–13.6)	3.28±0.11 (2.97–3.22)	36.3±0.44 (36.0–36.8)	7.27±0.21 (7.1–7.5)
*Ganoderma applanatum*	6.57±0.40 (6.1–6.8)	1.52±0.04 (1.48–1.56)	3.14±0.11 (3.02–3.22)	11.29±0.46 (10.9–11.8)	2.96±0.11 (2.84–3.06)	37.1±0.96 (36.0–37.8)	4.47±0.29 (4.3–4.8)

**Table 3.** Estimated daily intake of heavy metals (μg/kg/day) analysed in mushrooms collected during the field study.

Mushrooms	Lead		Cadmium		Chromium		Copper		Nickel		Zinc		Aluminum	
	AD	CHL	AD	CHL	AD	CHL	AD	CHL	AD	CHL	AD	CHL	AD	CHL
*Lenzites betulina*	3.54	10.33	0.69	2.03	1.80	5.26	7.69	22.44	2.48	7.23	57.60	168.00	3.47	10.13
*Pleurotus ostreatus*	2.71	7.91	0.48	1.41	2.06	6.01	1.83	5.33	0.72	2.10	9.27	27.03	1.83	5.35
*Calocybe indica*	3.46	10.08	0.53	1.55	1.35	3.93	2.73	7.98	0.93	2.73	12.30	35.87	1.59	4.65
*Pleurotus pulmonarius*	3.57	10.41	0.46	1.35	1.26	3.68	2.93	8.54	0.99	2.90	16.50	48.13	2.30	6.71
*Ganoderma sessile*	3.36	9.79	0.56	1.64	1.26	3.66	5.73	16.71	1.41	4.10	15.60	45.38	3.12	9.09
*Ganoderma applanatum*	2.82	8.21	0.65	1.90	1.35	3.93	4.84	14.11	1.27	3.70	15.90	46.38	1.92	5.59
PTDI [36]	NA		0.83		NA		500		NA		300 – 1000		140	

PTDI: Permissible tolerable daily intake; AD: Adult (70kg); CHL: Children (24kg); NA: Not available

**Table 4.** Target hazard quotient and hazard index of heavy metals in mushrooms

Mushrooms	Lead		Cadmium		Chromium		Copper		Nickel		Zinc		Aluminum		Hazard Index (∑THQ)	
	AD	CHL	AD	CHL	AD	CHL	AD	CHL	AD	CHL	AD	CHL	AD	CHL	AD	CHL
*Lenzites betulina*	0.89	2.58	0.69	2.03	0.60	1.75	0.19	0.56	0.12	0.36	0.19	0.56	0.0035	0.01	2.69	7.85
*Pleurotus ostreatus*	0.68	1.98	0.48	1.41	0.69	2.00	0.05	0.13	0.04	0.11	0.03	0.09	0.0018	0.0054	1.96	5.73
*Calocybe indica*	0.87	2.52	0.53	1.55	0.45	1.31	0.07	0.19	0.05	0.14	0.04	0.12	0.0016	0.0047	2.00	5.84
*Pleurotus pulmonarius*	0.89	2.60	0.46	1.35	0.42	1.23	0.07	0.21	0.05	0.15	0.06	0.16	0.0023	0.0067	1.96	5.71
*Ganoderma sessile*	0.84	2.45	0.56	1.64	0.42	1.22	0.14	0.42	0.07	0.21	0.05	0.15	0.0031	0.0091	2.09	6.09
*Ganoderma applanatum*	0.71	2.05	0.65	1.90	0.45	1.31	0.12	0.35	0.06	0.19	0.05	0.16	0.0019	0.0056	2.05	5.96

AD: Adult (70kg); CHL: Children (24kg)

**Table 5.** Carcinogenic risk of heavy metals in analyzed mushroom sample

Mushroom Sample	Pb		Cd		Cr		Ni		Tolerable limit
	AD	CHL	AD	CHL	AD	CHL	AD	CHL	
*L. betulina*	3.01E-05	8.78E-05	4.37E-03	1.27E-02	9.00E-04	2.63E-03	2.08E-03	6.07E-03	1E-06 - 1E-04
*P. ostreatus*	2.30E-05	6.72E-05	3.05E-03	8.88E-03	1.03E-03	3.01E-03	6.05E-04	1.76E-03	
*C. indica*	2.94E-05	8.57E-05	3.35E-03	9.76E-03	6.75E-04	1.97E-03	7.85E-04	2.29E-03	
*P. pulmonarius*	3.04E-05	8.85E-05	2.92E-03	8.51E-03	6.30E-04	1.84E-03	8.35E-04	2.44E-03	
*G. sessile*	2.86E-05	8.32E-05	3.53E-03	1.03E-02	6.30E-04	1.83E-03	1.18E-03	3.44E-03	
*G. applanatum*	2.40E-05	6.98E-05	4.10E-03	1.20E-02	6.75E-04	1.97E-03	1.07E-03	3.11E-03	

AD: Adult (70kg); CHL: Children (24kg)

**Table 6.** Data of metal concentrations (mg kg^-1^) in mushroom collected from Nigeria obtained from systematic review of published articles

References	Study location	Pb	Cd	Cr	Cu	Ni	Zn	Al	Co	Mn	Fe
[42]	Akure, Ondo	12.10	ND	ND	7.43	ND	15.31	ND	17.53	34.90	ND
[43]	Akure, Ondo	10.61	25.11	66.59	649.03	36.48	ND	ND	ND	ND	1051.19
[44]	Ife, Osun	ND	ND	0.62	ND	0.53	18.07	ND	0.23	ND	ND
[45]	Ife, Osun	ND	ND	0.44	0.54	0.35	34.86	ND	0.86	2.85	ND
[46]	Delta	ND	ND	0.70	5.07	3.95	ND	ND	ND	33.47	8.02
[47]	Edo State	ND	ND	ND	ND	ND	51.00	ND	ND	ND	442.00
[48]	Enugu	ND	ND	ND	7.00	ND	5.20	ND	ND	17.7	16.80
[6]	Rivers	0.51	0.22	ND	8.72	2.22	33.78	0.29	ND	ND	ND
[49]	Imo	0.13	ND	ND	0.164	0.0001	1.26	ND	ND	ND	ND
[50]	Imo	ND	ND	ND	1.86	ND	9.41	ND	ND	4.77	412.69
[51]	Akwa Ibom	0.86	0.25	ND	39.52	ND	90.81	ND	ND	27.77	505.07
[52]	Akwa Ibom	0.82	0.41	ND	29.92	0.97	47.27	ND	ND	29.09	ND
[53]	Bayelsa	3.75	0.20	0.60	5.43	ND	180.00	ND	0.81	28.5	584.75
[10]	Ibadan, Oyo	55.41	77.68	ND	75.04	ND	ND	ND	ND	ND	ND
[54]	Abia & Imo	0.74	0.048	0.26	6.20	0.43	23.00	18.00	0.049	18.00	52.00
[14]	Abia & Imo	1.35	0.79	2.55	3.51	2.14	25.56	ND	0.29	18.59	106.69
[55]	Rivers & Imo	ND	0.001	ND	0.07	ND	0.583	ND	ND	ND	1.82
[56]	Abia	1.88	0.83	ND	15.49	ND	51.02	ND	ND	ND	ND
[57]	Ebonyi	0.52	0.69	ND	ND	ND	ND	ND	ND	3.05	50.79
[58]	Abia	0.76	0.14	ND	ND	ND	ND	ND	ND	ND	74.50
[59]	Benin, Edo	1.49	3.47	ND	1.71	4.56	16.66	ND	ND	ND	21.44
Mean ± S.D		6.49±14.58	8.45±21.89	10.25±24.85	50.39±155.96	5.16±11.11	37.72±44.57	9.15±12.52	3.29±6.98	19.89±12.01	255.98±321.31

ND: Not detected according to respective author(s)

**Table 7.** Estimated Daily Intake, Target Hazard Quotient, Hazard Index and Carcinogenic Risk of heavy metals in mushroom samples from various states in Nigeria.

Heavy metals	EDI (μg/kg/day)		THQ = EDI/RfD_ingestion_		CR = EDI*SF	
	AD	CHL	AD	CHL	AD	CHL
Pb	2.78	8.11	0.695	2.03	2.36E-05	6.89E-05
Cd	3.62	10.56	3.62	10.56	2.28E-02	6.65E-02
Cr	4.39	12.81	1.46	4.27	2.19E-03	6.41E-03
Cu	21.59	62.99	0.54	1.57	ND	
Ni	2.21	6.45	0.11	0.32	1.85E-03	5.42E-03
Zn	16.17	47.15	0.054	0.16	ND	
Al	3.92	11.44	0.00392	0.011	ND	
Co	1.41	4.11	4.7	13.66	ND	
Mn	8.52	24.86	0.061	0.18	ND	
Fe	109.70	319.98	0.157	0.46	ND	
∑THQ (HI)			11.4	33.2		

ND: Not determined; RfDingestion: Oral Reference Dose; SF: Slope Factor of a carcinogen; AD: Adult (70kg); CHL: Children (24kg)

## List of Abbreviations

AAS: Atomic absorption spectrophotometer
CR: Carcinogenic risk
EDI: Estimated daily intake
FAO: Food and Agriculture Organisation
HI: Hazard index
HNO3: Hydrogen trioxonitrate (v) acid
PRISMA: Preferred Reporting Items for Systematic Reviews and Meta-Analyses
PTDI: Permissible Tolerable Daily Intake
RfD_ingestion_: Reference dose for each specific metal
SD: Standard Deviation
THQ: Target Hazard Quotient
WHO: World Health Organisation
